# Editorial: Domestication of Agronomic Traits in Legume Crops

**DOI:** 10.3389/fgene.2021.707600

**Published:** 2021-07-19

**Authors:** Xin Chen, Gaofeng Zhou, Jiayin Pang, Peerasak Srinives

**Affiliations:** ^1^Institute of Industrial Crops/Jiangsu Key Laboratory for Horticultural Crop Genetic Improvement, Jiangsu Academy of Agricultural Sciences, Nanjing, China; ^2^Department of Primary Industries and Regional Development, Government of Western Australia, South Perth, WA, Australia; ^3^The University of Western Australia Institute of Agriculture, University of Western Australia, Perth, WA, Australia; ^4^Department of Agronomy, Kasetsart University, Bangkok, Thailand

**Keywords:** legume crops, agronomic traits, domestication, genomics techniques, breeding

Legumes (*Fabaceae*) play an active role in food security, including maintain human health, mitigate climate change, and increase biodiversity. Legume crops are important source of human foods, animal feeds, and industrial materials. In human diets, legumes are among the primary sources of plant-based protein, and they are high in dietary fiber, as well as vitamins and minerals (Mudryj et al., 2014). Therefore, FAO emphasized and pointed out that eating legumes as part of a healthy diet can contribute to addressing the national nutritional problems, from undernutrition and micronutrient deficiencies to obesity and diet-related diseases, which exist in many countries. Leguminous crops are grown worldwide, making them important economically. Ensuring the grain yield and improving the quality of legumes are research goals of plant scientists. Evidence from archaeological and crop diversity research indicates that the domestication of major crops has multiple origin centers (Turrill, 1926). The domestication of legume crops greatly enriched the dietary structure of human beings and provided food security guaranteed for the reproduction and development of human civilization. Enhancing our understanding of the legume crops domestication and improvement process can help to better boost legume breeding efforts. This Research Topic included 19 original or review articles focused on multiple aspects of legume domestication, including but not limited to quantitative trait loci (QTL) mapping and association mapping of domestication-related genes, the function and interaction of domesticated genes, RNA-seq analysis of genes associated with agronomic and adaptive traits.

Domestication is a process in which people use existing experience or knowledge to turn wild animals and plants into controllable, and ultimately harvest products according to human needs. Therefore, crop domestication can also be seen as an evolutionary process of human intervention, and domestication genetics is an important branch of evolutionary biology research. Legume crops have been domesticated and used as parts of the human diet for more than 10,000 years ago, among which lentils (*Lens culinaris* Medik.), chickpeas (*Cicer arietinum* L.), and peas (*Pisum sativum* L.) are Neolithic founder crops. They were among the plant species domesticated by early farmers in the Fertile Crescent region (Calles et al., 2019). In the long cultivation history, the domesticated legumes gradually lost parts of the characteristics of their wild ancestors. While the characteristics addressing the needs of people have been accumulated and strengthened, and finally formed the modern cultivated species with high grain yield and good quality. It is documented that the domesticated legumes emphasized the selection of favorable traits, including but not limited to the reduced seed dormancy, larger seed size, palatability, root architecture, and root exudate composition (Abbo et al., 2014; Kim et al., 2021; Liu, Ku et al.). In this Research Topic, Lei et al. have investigated the genetic architecture of seed traits (100-seed weight, seed length, seed width, and seed height) of common bean (*Phaseolus vulgaris* L.) in China, and identified QTLs that could be used in marker-assisted breeding. The nitrogen fixation symbiosis in legumes is discussed in this topic (Liu, Yu et al. Marques et al.), showing that it is important for researchers to engineer or screen for elite rhizobia that are both competitive for nodulation and capable of high rates of nitrogen fixation. Another research in this topic reports soybean genotypes to have extremely low seed phytate concentrations, combined with important root traits for efficient phytate-phosphorus acquisition, providing material for soybean breeding (Kuerban et al.).

The domestication process has reduced the genetic diversity of the cultivated germplasm and self-sustaining capability, but increased susceptibility to pests, abiotic stresses and nutrient depletion in cultivated legumes, which can be illustrated by the research of soybean and common bean in this Research Topic (Nakata et al., Shi et al., Zhang et al. and Zhong et al.). The genetics of the traits, which are important for domestication, are studied in this topic. Wang et al. construct a single nucleotide polymorphism (SNP) genetic linkage map, which is good for precisely mapping genes, and is useful for investigating the mechanism of leaf development in mung bean or legumes. Hu et al. perform transcriptome and metabolome analyses, showing that the pod degreening of the golden hook in common bean resulting from chlorophyll degradation. Berger et al. reveal that the large wild-domestic differences (e.g., water extraction, water use efficiency and harvest index) between wild and domestic *Cicer* are indicative of evolution under contrasting selection pressures. In the study of Lee et al., a locus controlling compound raceme inflorescence in mung bean is identified, which provides valuable genetic information for understanding the architecture of the compound raceme in mung bean. Ku et al. discussed the effects of domestication on secondary metabolite composition in legumes. The authors pointed out that the genes responsible for determining the secondary metabolite composition that might have been lost due to domestication, and understanding these genes would enable breeding programs and metabolic engineering to produce legume varieties with favorable secondary metabolite profiles. Another study in this topic assesses the genetic diversity of common bean in Chongqing, China, providing support for the development of excellent germplasm and genetic resources (Long et al.).

At present, molecular genetics has been used to trace the evolutionary origin and domestication history of legume crops. Analytical research on the genome segments of crop domestication was undertaken mainly through population genetics analysis approaches based on genome resequencing and quantitative trait loci analysis based on linkage disequilibrium theory. The breakthroughs of legume crops made in recent years are mainly concentrated in soybean [*Glycine max* (L.) Merr.], common bean (*Phaseolus vulgaris* L.), rice bean (*Vigna umbellata*), and cowpea [*Vigna unguiculata* (L.)] (Jia et al., 2019). In this topic, QTL mapping, association mapping, and cloning of domesticated genes have given insight into domestication-related plant agronomic traits. Somta et al. and Amkul et al. identify QTLs controlling the agronomic traits in black gram [*Vigna mungo* (L.) Hepper] and zombi pea [*Vigna vexillata* (L.) A. Rich], respectively. Takahashi et al. performed fine-mapping in adzuki bean and yard-long bean, showing that independent domestication on the two legumes has selected the same locus for the non-shattering phenotypes. Another paper in this topic summarizes the known modifications in soybean circadian clock components as a result of domestication and improvement (Li and Lam).

In recent years, genome editing technology represented by CRISPR/Cas9 has been widely used in crops. At present, this technology is used to improve the wild relatives of crops in a targeted way to accelerate their domestication. With the deepening of the research, more domestication-related genes show high value in legume breeding. In addition, genomics techniques have been widely used in the study on the mechanism of legume crop domestication. Quantitative genetic parameters based on high-density SNP markers, such as diversity (π) and likelihood ratio (CLR), are found helpful in selection analysis. Significant diversity reduction parameter (π_wild_/π_cultivar_), population differentiation statistics *Fst*, and cross-population complete likelihood ratio (XP-CLR) approach were developed and used to identify selective sweeps between two groups (Shi and Lai, 2015). Nevertheless, the understanding of legume domestication is still very limited. The main problems to be solved are as follows: (1) The number of domestication genes cloned in legume crops is still very limited, and a large number of selection regions for domestication revealed by population genetic research still need further research. (2) Intensive genetic analysis of the cloned domesticated genes was undertaken, but research on the molecular regulation mechanism and regulation network pathway remains weak. (3) The research on legume crop domestication process mostly focuses on solving theoretical understanding problems, while the exploration of breeding and utilization approaches of related genes is still lacking.

## Author Contributions

All authors co-edited the Research Topic, wrote, edited, and approved the final version of editorial.

## Conflict of Interest

The authors declare that the research was conducted in the absence of any commercial or financial relationships that could be construed as a potential conflict of interest.
